# GABAergic inhibition of the nucleus reuniens stabilizes post-learning quiet states and enhances memory consolidation

**DOI:** 10.1093/sleepadvances/zpag108

**Published:** 2026-10-09

**Authors:** Luis M Guemarez, Gabriela Rico, Annie da Costa Souza, Carmen Varela

**Affiliations:** Department of Psychology, Florida State University, Tallahassee, FL, United States; Department of Psychology, Florida Atlantic University, Boca Raton, FL, United States; Department of Psychology, Florida State University, Tallahassee, FL, United States; Department of Psychology, Florida Atlantic University, Boca Raton, FL, United States; Department of Psychology, Florida State University, Tallahassee, FL, United States; Department of Psychology, Florida Atlantic University, Boca Raton, FL, United States

**Keywords:** learning and memory, sleep/wake cognition, sleep/wake mechanisms, pharmacology—sedative-hypnotics, psychiatric disorders

## Abstract

Quiet states provide a permissive context for processes that benefit from reduced engagement with the environment, such as memory consolidation, but the circuit mechanisms that stabilize these states remain unclear. The midline thalamic nucleus reuniens (RE) regulates hippocampal–cortical interactions during memory consolidation, but its role in stabilizing post-learning quiet states is unknown. Here, we tested the effects of RE inhibition on quiet-state stability and memory consolidation in freely behaving rats. Following object-place recognition training, rats received RE microinfusions of muscimol or vehicle and we monitored their behavior during the subsequent period. We found that RE inhibition increased the duration of quiet bouts and reduced bout fragmentation. Twenty-four hours later, muscimol-treated rats showed enhanced object–place recognition memory. These findings suggest that RE regulates the stability of quiet states that facilitate memory consolidation and provide a circuit mechanism linking thalamic inhibition, quiet states, and memory function.

## Teaser

**Statement of Significance** Disruption of quiet states and impaired memory are common features of many neurological and psychiatric disorders, but the mechanisms linking resting brain states to memory remain poorly understood. This study identifies the thalamic nucleus RE as a brain region that can regulate the stability of post-learning quiet states and influence memory consolidation.

## Introduction

Memory consolidation processes are critical to mental health as they enable mammals to integrate information from different experiences and develop mental models that support normal adaptive behavior.^1^^,^^2^ Memories are consolidated during states of reduced engagement with the environment, including quiet periods of wakefulness^3–6^ and sleep,^7^^,^^8^ which provide periods with reduced demands for information encoding.^9^ These states are associated with hippocampal memory reactivation in coordination with thalamocortical circuits, supporting memory stabilization and generalization.^7^^,^^10–12^ Consistent with the importance of these states for consolidation, disruption of memory consolidation is a common feature of mental health disorders associated with disrupted sleep^13^^,^^14^ and altered or insufficient periods of quiet wakefulness.^15^

Although the neural mechanisms that maintain the quiet states supporting memory consolidation remain unclear, the nucleus reuniens (RE) and the broader midline thalamus of which RE is part of, may play a central role. Midline thalamic neurons decrease their average firing rate around hippocampal sharp-wave ripples (SWRs; markers of episodic memory reactivation),^11^^,^^16^^,^^17^ and present an increased proportion of burst firing,^11^ which occurs when thalamic neurons are hyperpolarized.^18^ In addition, causal manipulation studies suggest that inhibition of the thalamus contributes to stabilizing quiet states and sleep.^19–22^ Consistent with this idea, pharmacological enhancement of GABAergic signaling is used clinically to promote sleep and treat insomnia.^23^^,^^24^ However, it remains unknown whether GABA agonists influence quiet-state stability through specific brain regions such as the midline thalamus and, in turn, enhance memory consolidation.

Within the midline thalamus, RE is particularly well positioned to mediate positive effects on memory. This nucleus is a critical hub linking the hippocampus and medial prefrontal cortex and contributes to memory consolidation during post-learning resting states,^25^^,^^26^ yet the mechanisms remain poorly understood.

Here, we tested whether pharmacological inhibition of RE with the GABAA receptor agonist muscimol^27^ alters post-learning quiet-state stability and memory consolidation in freely behaving rats. We focused on object–place recognition (OPR), a hippocampus-dependent task that also requires RE^28–30^ and depends on post-learning quiet states for memory consolidation.^28^^,^^31^^,^^32^

## Materials and methods

### Animals and surgery

Adult male and female Long–Evans rats (275–400 g) were implanted under sterile surgical conditions with a guide cannula targeting the nucleus RE of the thalamus for intracerebral drug microinfusion. Because the paired RE nuclei lie along the midline, a single-guide cannula was sufficient to target both nuclei. Stainless-steel guide cannulas (26 G; Protech International) were implanted 2 mm above RE (P1.8 mm, L1.8 mm, and depth 4.5 mm from Bregma) and secured with dental acrylic and skull screws. A dummy cannula remained in place except during drug infusion. Injection needles used for infusion extended 2 mm beyond the guide cannula to reach RE. A small magnet embedded in the dental acrylic allowed the attachment of a 5 mm red light-emitting diode (LED) used for video-based tracking of head position during the experiments.

All animal procedures were approved by the Institutional Animal Care and Use Committees of Florida Atlantic University and Florida State University and were conducted in accordance with the National Institutes of Health Guide for the Care and Use of Laboratory Animals.

### Behavioral procedures

Following postoperative recovery, rats were habituated to handling by the experimenter and to an enclosed recording arena (“rest arena”) during three to five daily daytime sessions. Rats were left undisturbed in the rest arena, which was later used to record spontaneous post-learning behavior following RE infusions. Memory was assessed using an OPR task, which consisted of a 10-min training session and a subsequent 5-min test 24 h later. During training, rats explored two identical objects placed in opposite corners of a square “OPR arena” with fixed visual cues on the walls. During the 24-h test, one object was moved to a novel location while the other remained stationary. Memory performance was quantified using object exploration times, the fraction of time spent exploring the moved object, and a discrimination index (DI), with higher positive values indicating greater preferential exploration of the moved object and stronger memory for the original object locations.

Behavioral testing was conducted during the light phase (ZT0–ZT12) with training beginning at ZT 3.96 ± 0.35 h (mean ± SEM) in the muscimol group and ZT 4.00 ± 0.31 h in the vehicle group; post-training rest began at ZT 4.57 ± 0.32 h and ZT 4.57 ± 0.30 h, respectively; and the memory test began at ZT 3.58 ± 0.42 h and ZT 3.69 ± 0.43 h, respectively. No differences were observed for any (Wilcoxon signed-rank test; Holm-adjusted *p* = 1.0 for all comparisons).

### Inclusion and exclusion criteria and statistics

We initially tested 13 rats. Two animals were excluded from memory analyses because they failed to meet the predefined criterion of at least 5 s of object exploration for each object in every training and test session. This threshold reflects our conservative definition of object exploration, which required rats to actively orient toward and sniff the object. Video recordings were unavailable for quiet state analyses in one session from each of two rats due to technical issues. Accordingly, quiet state analyses included all available sessions and were analyzed with Wilcoxon rank sum tests, whereas memory analyses were based on paired data and we used Wilcoxon signed-rank tests. For analysis across training and test sessions, we used 2 × 2 repeated-measures Analysis of Variance (ANOVA) with treatment (vehicle, muscimol) and session (training, test) as within-subject factors. Holm–Bonferroni correction was applied for multiple comparisons where appropriate.

### Drug preparation and microinfusion

Muscimol (cat # M1523 from Sigma, and conjugated muscimol from Fisher Scientific—cat # M23400—in one rat) was dissolved in sterile phosphate-buffered saline (PBS; pH 7.4) at a concentration of 1 μg/μL, aliquoted, and stored at −80°C until use. The muscimol dose was selected based on previous inactivation studies in behaving rats and to provide effective RE inhibition during the post-learning period.^33^^,^^34^ Muscimol or vehicle (PBS) was infused into RE at 0.2 μL/min for a total volume of 0.5 μL. Injection needles extended 2 mm beyond the guide cannula to reach RE and remained in place for >1 min after infusion to facilitate drug diffusion in the tissue. Rats were then returned to the rest arena, where periods of quiet and active wake behavior were monitored continuously for 4–5 h.

### Monitoring of active and quiet states

Animal position was tracked using open-source Bonsai software by monitoring the red LED mounted on the head implant. Quiet bouts were identified when speed remained below 2 cm/s for at least 2 min. Quiet state stability was quantified from the number of resting bouts and mean bout duration.

### Electrophysiological validation

Two additional rats implanted with tetrodes in hippocampal CA1 and neocortex (using methods described in^11^) were used for methodological validation. In one animal, a guide cannula implanted in RE and tetrodes in the medial prefrontal cortex allowed assessment of the effect of RE muscimol infusion on cortical slow oscillation (SO; delta band, 1–4 Hz) activity (Figure 1D and E). We detected SOs as local minima in the delta-filtered local field potential (LFP) that exceeded an amplitude threshold based on the mean and standard deviation of the delta-filtered LFP during quiet states. In the second animal, with tetrodes in the retrosplenial cortex and hippocampus, video-based detection of resting bouts was compared with electrophysiological recordings of cortical delta and hippocampal sharp-wave ripple (100–275 Hz) power, demonstrating close agreement between the two methods (Figure 1F and G).

**Figure 1:**
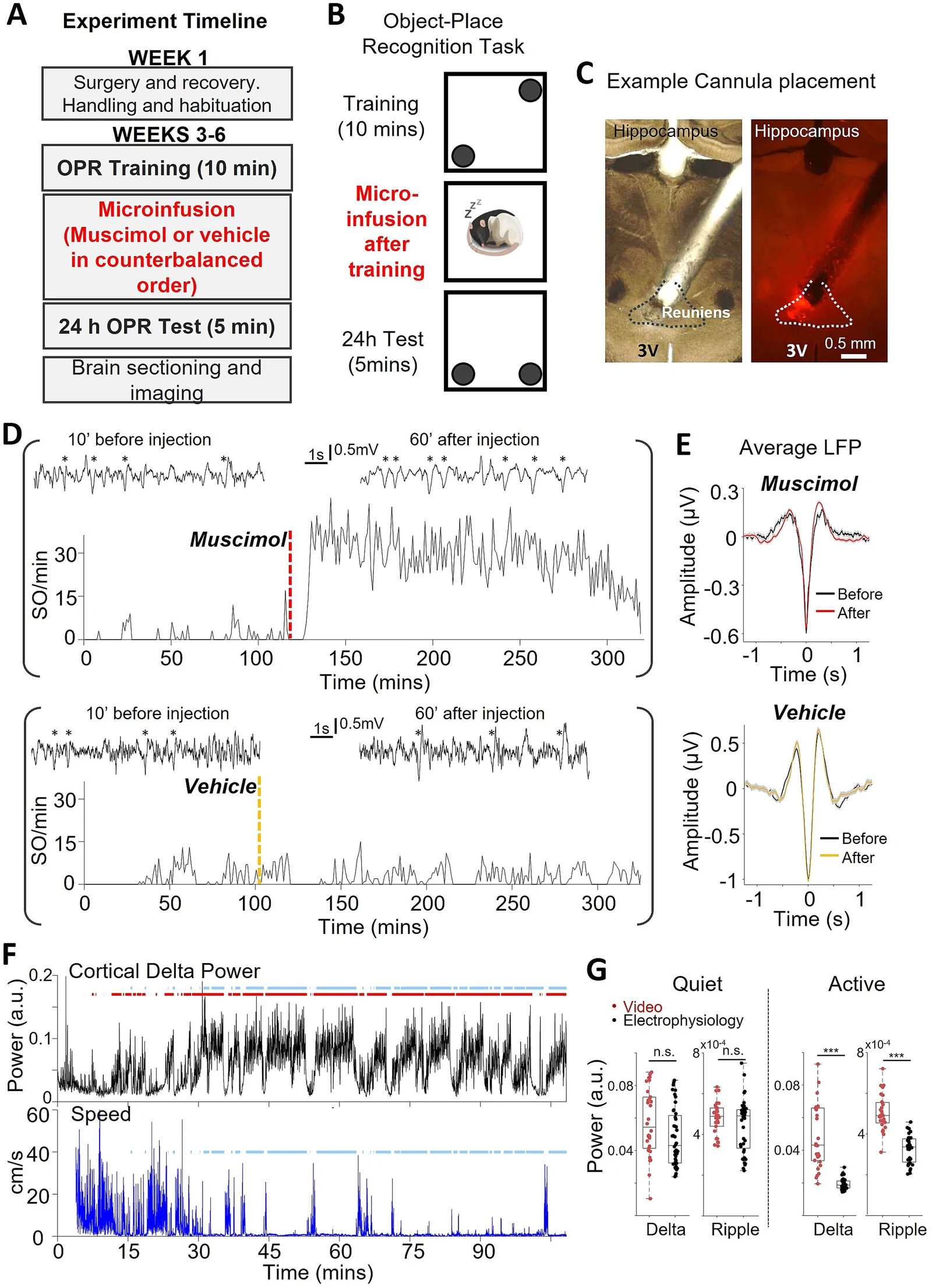
Experimental design and electrophysiology approach validation. (A) Experiment timeline and (B) behavioral task. Rats underwent object-place recognition (OPR) training followed immediately by microinfusion of muscimol (500 nL, 1 μg/μL in PBS; 0.2 μL/min) or vehicle (500 nL PBS, pH 7.4; 0.2 μL/min) into the nucleus reuniens (RE) followed by 4–5 h of post-training monitoring of quiet states before OPR testing 24 h later. (C) Representative coronal section showing the infusion cannula track in RE; fluorescently tagged muscimol was infused in this rat; left, light-microscopy image, right, red-channel confocal image. 3V, third ventricle. (D) Cortical slow-oscillation (SO) rate following muscimol (top) and vehicle (bottom) infusion during separate recording sessions in the same rat as in (C). Representative raw LFP traces before and after infusion are shown above the average SO rate. Vertical dashed lines indicate infusion time. (E) Average cortical LFP traces of SOs centered on the oscillation trough before and after muscimol (top) and vehicle (bottom) infusion. (F) Cortical LFP delta power (top) and movement speed (bottom) with cyan horizontal bars indicating quiet-state bouts identified by the video-based algorithm based on low speed; red bars indicate bouts identified using electrophysiological criteria. G) Cortical delta and hippocampal ripple power during quiet-state bouts (left) and active bouts (right) identified by video-based movement tracking or electrophysiological criteria. Quiet bouts identified by the two methods showed similar delta and ripple power (Holm-adjusted *p* > .05, Wilcoxon rank-sum tests), whereas video-identified active bouts had higher delta and ripple power than electrophysiology-defined active bouts (*p* < .001, Holm-adjusted, Wilcoxon rank-sum tests).

## Results

Quiet states in the hours that follow episodic memory acquisition contribute strongly to its consolidation, including OPR memory.^28^^,^^31^^,^^35^ To test whether transient inhibition of RE promotes quiet states and episodic memory consolidation, we used local muscimol microinfusion in RE right after training rats in a standard OPR task. After recovery from implant surgery and habituation to the experimental context (Figure 1A), rats underwent OPR training, encoding the locations of two identical objects during a 10-min training session when they freely explored the enclosure with the objects. Animals received an RE infusion of muscimol (0.5 μL, 1 μg/μL in PBS; 0.2 μL/min) or vehicle (0.5 μL PBS; 0.2 μL/min) immediately after training and were then placed in a familiar enclosed “rest arena,” where spontaneous behavior was video-monitored continuously for 4–5 h. We tested memory 24 h later with a 5-min test in the same OPR arena, where one object was displaced to a novel location. We selected a 24-h retention interval to minimize acute effects of muscimol on retrieval and assess long-term memory consolidation. Figure 1C shows an example of the infusion cannula targeting, and Figure S1 has a diagram with infusion sites for all the animals. In a separate validation experiment in a rat implanted with an RE microcannula and cortical electrodes (see Materials and Methods), we characterized the time course of the muscimol effect compared to vehicle infusion on cortical SOs, which are associated with quiet states.^8^^,^^36^ This recording showed that muscimol infusion in RE was associated with an increase in the occurrence of cortical SOs (Figure 1D), without altering their waveform shape or amplitude (Figure 1E).

To quantify the effect of RE muscimol infusion on quiet states we used a video-based algorithm (Materials and Methods) that identified periods of immobility while the rats were in the rest arena. We did not attempt to separate quiet wakefulness from sleep, as our goal was to characterize how RE inhibition influences general quiet states that support memory reactivation and hippocampal–cortical communication, which occur during immobility and are not limited to sleep.^6^^,^^10^^,^^12^^,^^37^ We validated the video detection of quiet states against electrophysiological scoring in an independent animal (Figure 1F and G). Of the electrophysiology-defined quiet bouts, 70.5% were detected by video analysis with start times within 2 min, and 59% also had endpoints within 2 min. Video-identified quiet bouts had similar cortical delta and hippocampal ripple power to electrophysiology-defined resting bouts (Holm-adjusted *p* > .05, Wilcoxon rank-sum test). Although our study focuses on quiet states, we also examined active bouts identified by video-based movement tracking. These bouts showed higher delta and ripple power than electrophysiology-defined active bouts (*p* < .001, Holm-adjusted, Wilcoxon rank-sum test). Ripple activity is well documented during wakefulness,^38^^,^^39^ and delta activity has also been reported in cortical areas in quiet wakefulness.^36^ The validation recording was performed in an enclosure that was familiar to the animal and had no objects or attentional demands, which may have contributed to the presence of delta and ripple activity during active bouts in this context compared with behavioral tasks that might have involved external stimuli and attentional demands. Overall, these results support the use of video-based movement tracking to identify quiet bouts corresponding to behavioral rest with oscillatory features associated with low-arousal and sleep states (Figure 1G).

We then calculated the number of quiet bouts and their duration (Figure 2) after pharmacological infusion and found a significant increase in bout duration (mean 82.33 %; *n* = 11) following muscimol compared to vehicle infusions (*p* = .036, Wilcoxon rank sum test) and a decrease in the total number of bouts (mean 18.74%; *n* = 11; *p* = .021, Wilcoxon rank sum test), while the session duration was not significantly different between muscimol and vehicle (*p* = .7; Wilcoxon rank sum test).

**Figure 2:**
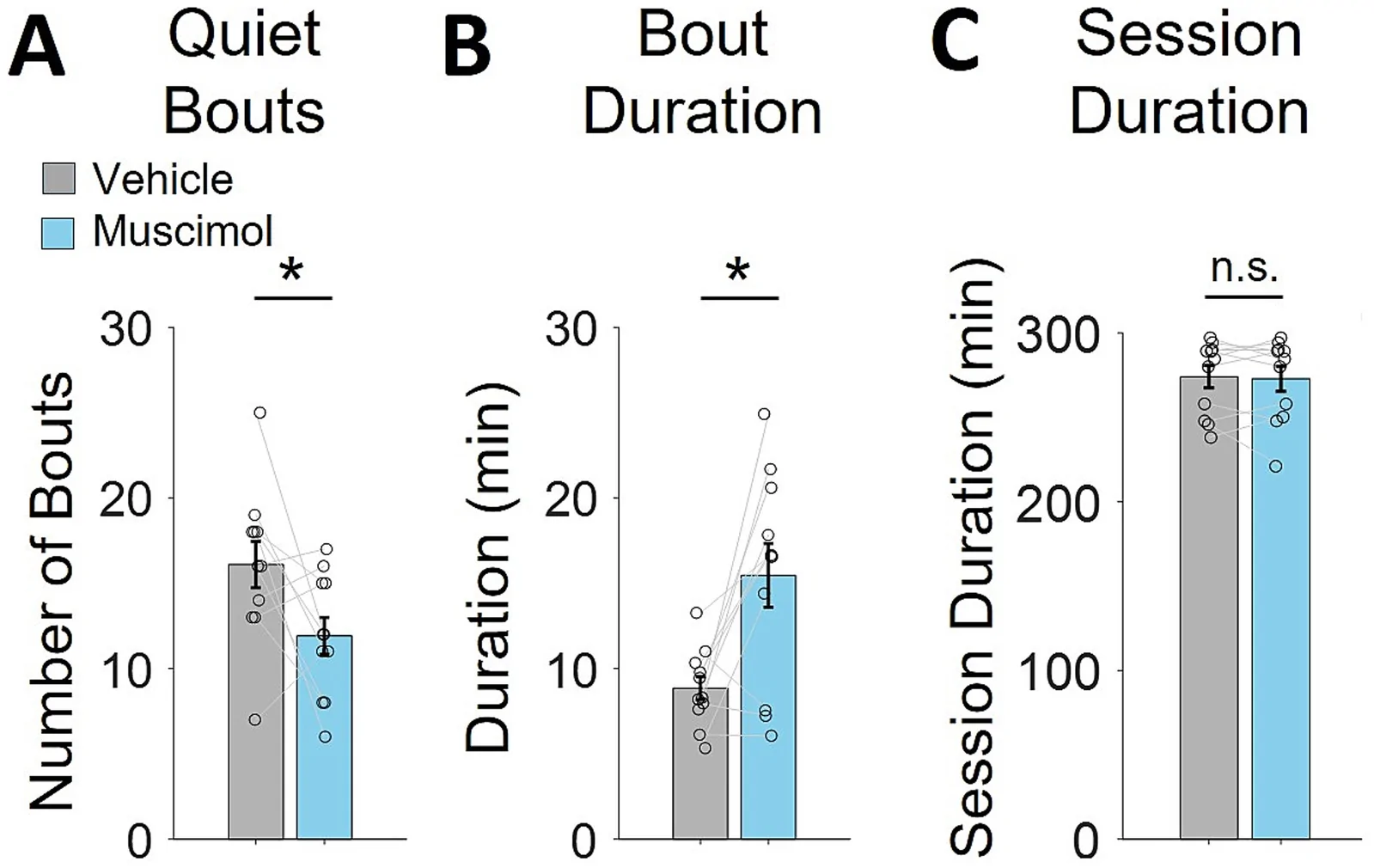
Muscimol microinfusion in reuniens increases post-learning quiet state stability. (A) Number of quiet bouts during the post-training recording session following vehicle or muscimol infusion in reuniens. (B) Mean quiet-bout duration. (C) Total session duration. Bars indicate mean ± SEM; circles represent individual animals, *n* = 11. Statistical comparisons were based on Wilcoxon rank sum tests, ^*^  *p* < .05.

Quiet states facilitate the consolidation of episodic memories, including in the OPR task.^28^^,^^31^^,^^32^ We analyzed memory consolidation in within-animal experiments in which each rat was tested in the muscimol and vehicle condition (order counterbalanced across rats) at least 1 week apart; we used different objects and visual cues in the two conditions to promote distinct memory experiences. As measures of memory, we calculated the proportion of time rats spent exploring the moved compared to the stationary object and a DI between the two objects during training and the 24 h-test, compared to the same rats when they received vehicle post-training. We performed a 2 × 2 repeated-measures ANOVA with treatment (vehicle, muscimol) and session (training, test) as within-subject factors. We examined data distributions and model residuals to confirm that normality assumptions were satisfied. The analysis revealed a significant main effect of session on the fraction of time exploring the moved object (Figure 3A; Figure S2 includes analyses of total exploration time for the moved and stationary objects) and DI (Figure 3B), with both metrics being higher in the 24-h test (*F* [1, 10] = 7.37, *p* = .022), suggesting memory consolidation but no significant main effect of treatment or treatment × session interaction, suggesting that the magnitude of the 24-h effects did not differ in muscimol compared to vehicle. Specifically, in the 24-h test, rats spent proportionally more time exploring the moved object following muscimol (61.66 ± 3.66 %) and vehicle (53.63 ± 3.66 %) and had higher DI values (muscimol, 0.233 ± 0.073; vehicle, 0.073 ± 0.073), but the higher values observed in muscimol compared to vehicle did not reach significance after Holm correction (uncorrected *p* = .032, Wilcoxon signed-rank test; Holm-adjusted *p* = .056). Because the vehicle condition reflects the baseline memory consolidation, we additionally considered whether the change in DI from training to the 24-h test differed from zero within each treatment condition. The DI increased significantly following muscimol (mean change ± SEM 0.29 ± 0.11, Holm-adjusted *p* = .037, one-sample Wilcoxon signed-rank test), whereas the vehicle condition showed a positive but non-significant change (0.17 ± 0.11, Holm-adjusted *p* = .102).

**Figure 3:**
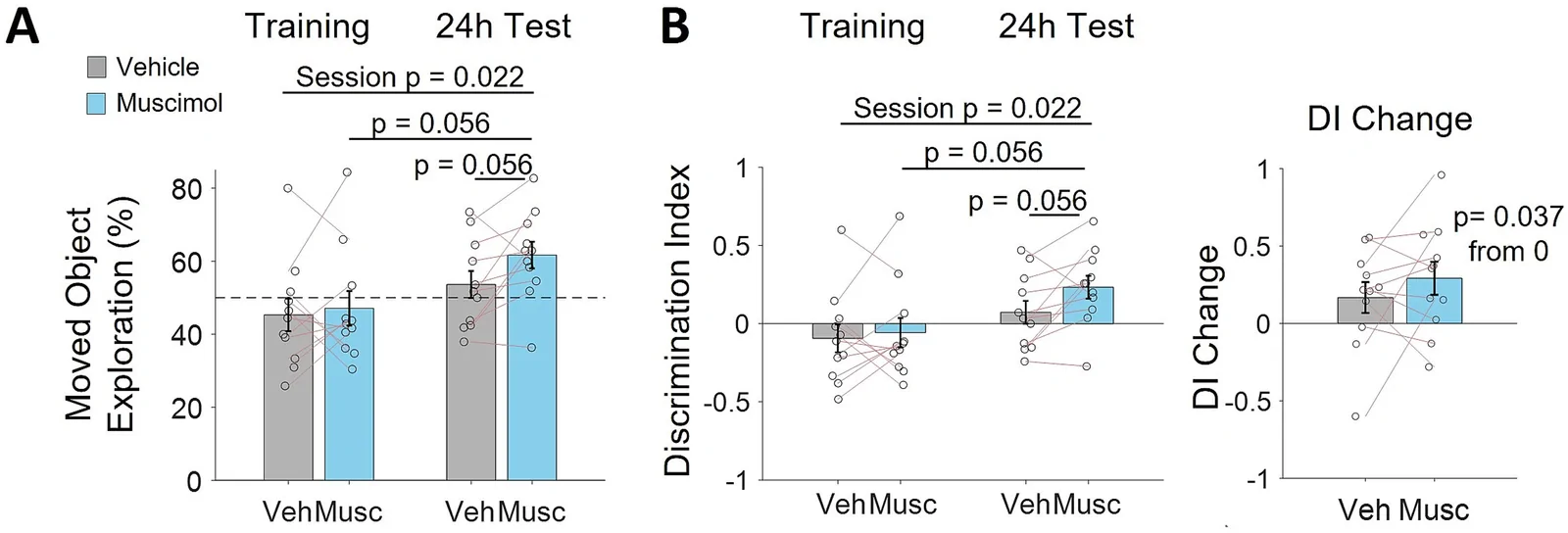
Muscimol in reuniens enhances object-place memory consolidation. (A) Fraction of exploration time spent on the moved relative to the stationary object during training and the 24-h test following vehicle or muscimol infusion in reuniens. (B) Discrimination index (DI) during training and the 24-h test under vehicle or muscimol conditions. A significant main effect of session was observed for both the % exploration of the moved object and DI (*F* [1, 10] = 7.37, *p* = .022). Rats also showed a significant increase in DI from training to test relative to zero (Holm-adjusted *p* = .037, one-sample Wilcoxon signed-rank test). Bars indicate mean ± SEM; circles represent individual rats, *n* = 11. *p*-values on the plots are based on the 2 × 2 repeated-measures ANOVA or paired/one-sample Wilcoxon signed-rank tests with Holm correction.

Together, these findings indicate that rats showed a significant training-to-test increase in object DI following muscimol infusion in RE, although the magnitude of this change did not differ significantly from that observed following vehicle, consistent with memory consolidation that may also occur under baseline conditions.

We also used linear regression to test whether the improvement in memory (the change in the DI in muscimol compared to vehicle) was linearly correlated with the increase in quiet bout duration or the number of bouts, but we did not find significant correlations (Figure S3; *r*2 < 0.01; *p* > .4). Altogether these results suggest that muscimol-mediated inhibition of RE promotes stable quiet states that support episodic memory consolidation. Interestingly, the rats did not show evidence of a strong memory trace in the vehicle 24-h test, suggesting they had formed a weak memory that faded within a day, and the results demonstrate that our pharmacological intervention to promote quiet states following encoding was sufficient to enhance a memory that would normally have decayed.

Some studies report sex differences in spatial memory consolidation, but sex-specific analyses remain scarce in the literature.^40^^,^^41^ We therefore performed a preliminary analysis separating our sample by sex to identify potential sex-dependent effects of RE inhibition. Despite the small sample size (seven males, four females), we found that the main results shown in the previous figures were consistent across sexes (Figure 4). In particular, in the 24-h memory test, both males and females showed higher exploration times of the moved compared to the stationary object following muscimol infusion (males spent 54.93% more time on the moved object and females 151.83% more time), compared to after vehicle (26.04% more time exploring the moved object in males, 43.95% more time in females). Consistent with the exploration times, both males and females showed an increase in DI following muscimol (Figure 4B), and the increase was 38% larger in females than males (mean DI increase was 0.26 in males, 0.35 in females). In comparison, the mean DI change after vehicle was much smaller and similar in both sexes (mean DI increase by 0.18 in males and by 0.15 in females).

**Figure 4:**
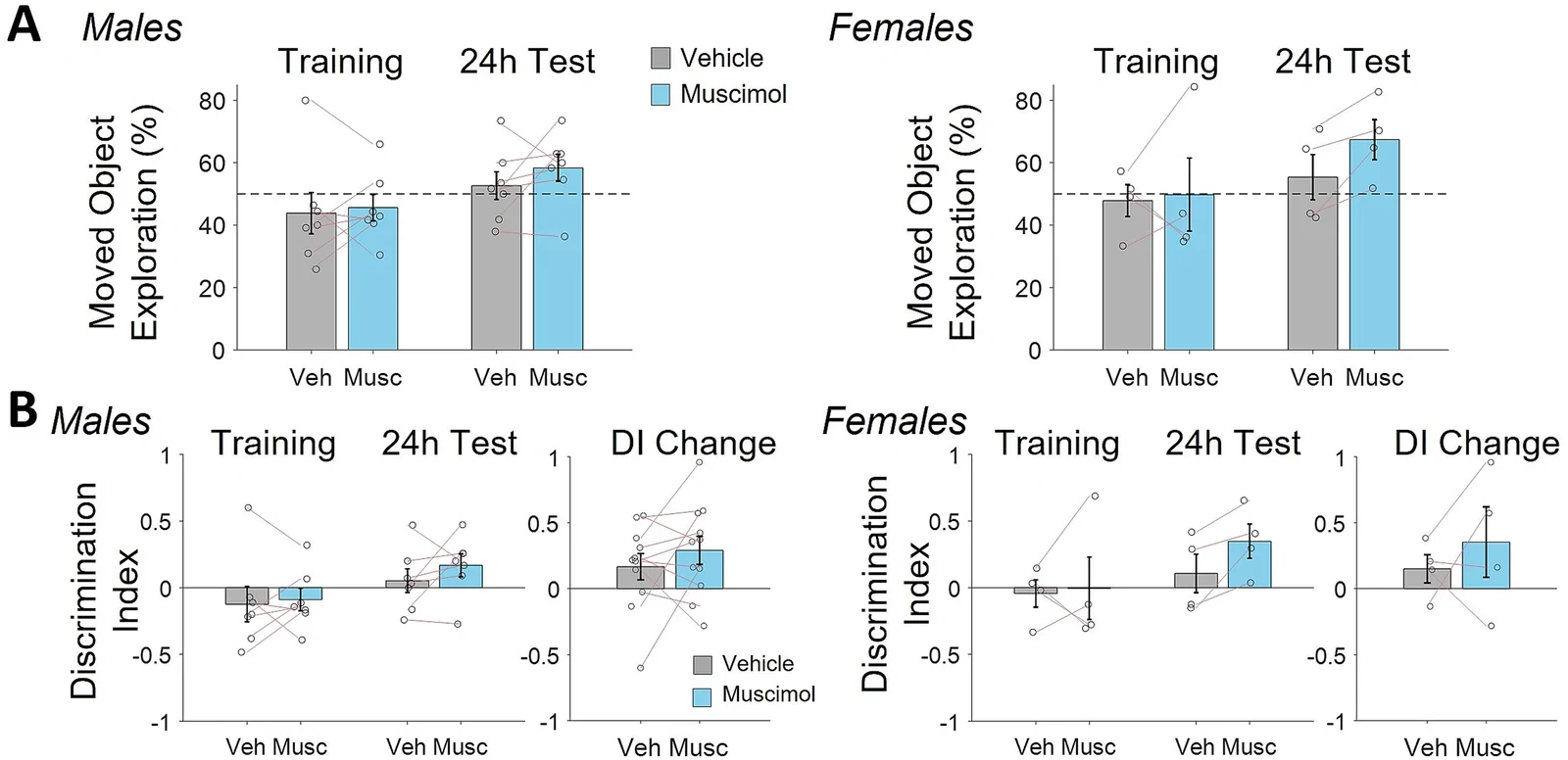
Analysis of muscimol effect on object–place memory consolidation by sex. (A) Fraction of exploration time of the moved relative to the stationary object during the training and 24-h tests for male (left panel) and female (right) rats following vehicle or muscimol infusion into reuniens. (B) Discrimination index (DI) during training and the 24-h test shown separately for males and females. In both sexes, muscimol was associated with greater values of preferential exploration of the moved object and DI at the 24-h test compared with vehicle. Bars indicate mean ± SEM; circles represent individual rats. Statistical tests based on 2 × 2 repeated-measures ANOVA and paired or one-sample Wilcoxon signed-rank tests, with Holm correction for multiple comparisons, revealed no significant differences after correction.

We performed exploratory statistical analyses in the male and female groups using 2 × 2 repeated-measures ANOVA. Although the male analysis showed a nominal increase in the relative exploration time of the moved object from training to the 24-h test following muscimol, this effect did not remain significant after correction (uncorrected *p* = .047, Wilcoxon signed-rank test; adjusted *p* = .188). No significant effects of session, treatment, treatment × session interaction, or difference from zero were detected in either males or females (*p* > .05 for all comparisons). Given the limited sample sizes, these sex-specific analyses should be interpreted cautiously, although the observed trends may provide a useful basis for comparison in future studies. While preliminary, these results suggest that muscimol produced effects in both sexes, with a trend toward a stronger effect in females. Interestingly, females also seemed to retain the memory better at 24 h after vehicle, whereas the weaker baseline memory in males raises the possibility that the effect of muscimol may depend in part on the strength of the underlying memory trace.

## Discussion

We found that acute infusion of muscimol in RE increased quiet-bout duration while reducing the number of quiet bouts and improving memory performance 24 h later. Our results suggest that RE inhibition is sufficient to promote more prolonged quiet states that support episodic memory consolidation. These findings identify a novel circuit mechanism linking quiet-state stability to memory consolidation and cognitive function, with potential translational relevance to neuropsychiatric disorders characterized by disruptions in quiet wakefulness^36^ and sleep.^13^^,^^14^

The mechanisms underlying these findings may involve complementary processes. First, longer, less fragmented quiet periods after learning may create a more stable window for consolidation, allowing hippocampal memory reactivation to proceed with fewer interruptions and increasing the opportunity for cortical stabilization of the reactivated memories.^35^ Consistent with our findings, enhancing activity in the thalamic reticular nucleus, the main source of GABAergic inhibition to the thalamus, promotes stable sleep,^20^^,^^22^ and our results raise the possibility that RE may partly mediate this effect. Second, our previous work showed that RE neurons are more likely to fire spikes in bursts (a signature of thalamic hyperpolarization) around the time of hippocampal SWRs.^11^ Inhibition of RE may promote stable quiet states through a switch to a burst firing mode. Consistent with that possibility, a selective knockout of CaV3.1 channels (which mediate burst firing in dorsal thalamus) in the midline thalamus increased sleep fragmentation in mice.^19^ Lastly, RE has been proposed to facilitate the temporal coordination between the hippocampus and the neocortex for systems-level memory consolidation^7^^,^^11^ and RE inhibition may further enhance consolidation by promoting hippocampal–cortical communication during quiet states.^4^

It is also important to consider that the increase in quiet bout duration did not significantly correlate with the magnitude of memory improvement. This lack of a linear relationship does not necessarily argue against a role for quiet-state stability in consolidation. Quiet-state stabilization may provide a permissive condition for consolidation rather than determining the magnitude of memory improvement. That is, longer and less fragmented quiet periods can provide greater opportunity for consolidation,^9^ while the efficacy of these processes may depend on the amount of hippocampal memory reactivation within that state and the degree of coordination between the hippocampus and the neocortex.^9^^,^^42^ Muscimol infusion in RE may not directly increase memory replay rate, as this is initiated within hippocampus,^43^^,^^44^ in line with the hypothesis that hippocampal and thalamic circuits may separately promote memory replay and the quiet states in which it can occur.

Understanding how the activation of GABA receptors via muscimol influences quiet bouts can have implications for understanding how pharmacological drugs that target these receptors might influence several interdependent functions that are supported by resting states, including not only memory processes but also metabolic, hormonal, and immune functions. Future studies incorporating physiological biomarkers could provide further useful insights into how quiet states (rest, sleep) influence health and behavior.

Our findings differ from a previous study that reported impaired OPR memory following post-training muscimol infusion in RE when animals were tested 3 h later.^29^ One possible explanation is that the 3-h interval may reflect both memory and residual pharmacological effects on retrieval,^45–47^ whereas the 24-h delay used here is more likely to isolate effects on memory consolidation. In agreement with our findings, microinfusion of muscimol in RE after acquisition of a fear memory in rats led to increased fear memory and generalization in a 24-h test, and the muscimol infusion decreased immediate early gene expression, suggesting that it reduced average neural activity in RE.^48^

Our findings also have methodological implications for studies using muscimol to transiently inactivate RE. Because RE inhibition promoted longer, less fragmented quiet states, these changes may interfere with cognitive performance. Pharmacological inhibition is frequently used to infer the contribution of a brain region to cognitive behavior, and monitoring resting behavior may therefore help interpret the behavioral consequences of RE inactivation.

Limitations of this study include that our behavioral approach quantified quiet states but did not distinguish quiet wakefulness from NREM sleep. However, this approach was designed to capture the broader post-learning states associated with hippocampal reactivation and memory consolidation, which include quiet wakefulness and sleep.^4^^,^^6^^,^^8^ The sex-specific analyses were exploratory and were not designed or powered to examine this factor and should therefore be interpreted as hypothesis-generating. Estrous cycle and potential sex-dependent differences in circadian and sleep physiology were not controlled, and these variables could contribute to the observed differences in quiet bouts and memory.^40^^,^^41^ Future studies with larger cohorts, additional sex-dependent metrics, and direct electrophysiological state classification will be needed to further define the mechanisms underlying RE-mediated regulation of quiet states and memory consolidation.

Together, our findings identify RE as a circuit through which GABAergic inhibition can stabilize post-learning quiet states to promote episodic memory consolidation. This mechanism may be particularly relevant to psychiatric disorders such as schizophrenia, post-traumatic stress disorder, and attention-deficit/hyperactivity disorder, in which thalamic dysfunction, quiet state abnormalities, and episodic memory deficits frequently coexist.^13^^,^^14^^,^^36^^,^^49–51^

## Supplementary Material

Supplementary_material_zpag108

## Data Availability

The data supporting the findings are available from the corresponding author upon reasonable request.
