# Supplementary material for: GABAergic inhibition of the nucleus reuniens stabilizes post-learning quiet states and enhances memory consolidation
Source: Sleep Adv. 2026 Oct 9;7(4):zpag108. doi: 10.1093/sleepadvances/zpag108 (PMC13750365; doi:10.1093/sleepadvances/zpag108)
Supplement: Supplementary file 1 [file supplementary_material_zpag108.docx]

**GABAergic inhibition of the nucleus reuniens stabilizes post-learning quiet states and enhances memory consolidation**

**Authors**: Luis M. Guemarez^1^, Gabriela Rico^2^, Annie da Costa Souza^1,2^, Carmen Varela^1,2^

**Author Affiliations:**

(1) Department of Psychology, Florida State University. 1107 W. Call Street

Tallahassee, FL 32306-4301. United States.

(2) Department of Psychology, Florida Atlantic University. 777 Glades Rd, SE-43

Boca Raton, FL 33431. United States.

**Corresponding Author**: Carmen Varela, PhD. Department of Psychology, Florida State University. 1107 W. Call Street. Tallahassee, FL 32306-4301. United States. [c.varela@fsu.edu](mailto:c.varela@fsu.edu)

**Supplementary Material**

**Figure S1**.

**Re**

Rt

PV

AV

AM

MD

IMD

Rh

**Re**

CM

1 mm

PV

Bregma P1.32mm

Bregma P2.04mm

CM

**Figure S1. Anatomical location of infusion sites.** Schematic representation of midline thalamic nuclei at two rostrocaudal levels showing the locations of infusion sites for all pharmacology animals. Diagrams are based on the Paxinos & Watson (1986) rat brain atlas. Abbreviations: Re, reuniens; CM, central medial; MD, mediodorsal; IMD, inter-mediodorsal; PV, paraventricular; AV, anteroventral; AM, anteromedial; Rh, rhomboid; Rt, thalamic reticular nucleus.

**Figure S2.**

**24h Test**

**Training**

**A**

St

Mo

St

Mo

St

Mo

St

Mo

Muscimol

Vehicle

*Males*

*Females*

St

Mo

St

Mo

Muscimol

Vehicle

Muscimol

Vehicle

St

Mo

St

Mo

St

Mo

St

Mo

**B**

**C**

Muscimol

Vehicle

Session p = 0.039

p = 0.039

p = 0.018

p = 0.039

St

Mo

St

Mo

Session p = 0.016

***Figure S2. (A)*** Absolute exploration time for the stationary and moved objects during training and the 24 h test. Across treatment groups, the pattern of object exploration changed from training to test (Session × Object interaction F(1,10) = 5.63, p = 0.039). Follow-up comparisons showed reduced exploration of the stationary object from training to test following both vehicle and muscimol, and greater exploration of the moved object than the stationary object at the 24 h test following muscimol. All *p*-values are Holm-corrected; paired comparisons used Wilcoxon signed-rank tests. Bars indicate mean ± SEM; circles represent individual rats connected by paired lines; *n* = 11. Panels (B) and (C) show the same analyses for males and females, respectively. The only significant finding was a main effect of session in females (F(1,3) = 23.96, p = 0.016).

**Figure S3.**

**Figure S3. Relationship between quiet states and memory improvement**. Linear regression between the change in discrimination index (DI; muscimol relative to vehicle) and changes in quiet bouts (left) or bouts duration (right). Neither relationship reached statistical significance.
